# Comparing Two Different Doses of Intravenous Ondansetron With Placebo on Attenuation of Spinal-induced Hypotension and Shivering

**DOI:** 10.5812/aapm.12055

**Published:** 2014-03-18

**Authors:** Seyed Mojtaba Marashi, Saeid Soltani-Omid, Sussan Soltani Mohammadi, Yasaman Aghajani, Ali Movafegh

**Affiliations:** 1 Department of Anesthesiology, Dr. Shariati Hospital, Tehran University of Medical Sciences, Tehran, Iran

**Keywords:** Serotonin, Heart Rate, Hypotension, Arterial Pressure, Ondansetron, Anesthesia, Spinal

## Abstract

**Background:**

Side effects of spinal anesthesia are hypotension, bradycardia and shivering. Five-hydroxytriptamine (5-HT), a serotonergic receptor, may be an important factor associated with inducing the Bezold Jarish reflex (BJR) that may lead to the bradycardia and hypotension in the setting of decreased blood volume.

**Objectives:**

This study aimed to investigate the effect of intravenous administration of ondansetron, a 5-HT3 receptor antagonist, which could attenuate spinal-induced hypotension, bradycardia and shivering.

**Patients and Methods:**

Two hundred and ten patients aged 20-50 years old were scheduled for spinal anesthesia and were divided randomly into three equal groups. The control group received normal saline and intervention groups received 6 mg or 12 mg of intravenous ondansetron 5 minutes before spinal anesthesia. Mean arterial pressure (MAP), heart rate (HR), and shivering were recorded before and after spinal anesthesia every 5 minutes during first 20 minutes of surgery.

**Results:**

Demographic data were not statistically different among groups. HR was statistically different between the experimental groups and the control group. Ten patients (14%) in the control group had HR < 50 bpm, that required intravenous atropine compared to experimental groups (P =0.02). In the control group 12 (17%) patients had MAP < 80 mm Hg and required vasopressors compared to experimental groups (P = 0.04). There were no significant differences in MAP and HR between the experimental groups (P =0.06). Incidence of shivering in the control group was 45% (32.70) that was statistically more than experimental groups (P = 0.02).

**Conclusions:**

Administration of two different doses of intravenous ondansetron, 6 mg and 12 mg, significantly attenuates spinal induced hypotension, bradycardia and shivering compared to the control saline group. However, the hemodynamic profiles and shivering in experimental groups were not statistically different.

## 1. Background

Spinal anesthesia is one of the common methods of providing anesthesia for various surgeries. Despite the popularity and ease of its use, this procedure is frequently associated with hemodynamic instability. The incidence of hypotension and bradycardia in non-obstetric patients has been reported to be 33% and 13%, respectively. In obstetric, non-laboring patients, the incidence of hypotension has been estimated to be as high as 50–60%; this is less common after the onset of labor (1-2). Probably reduction in vascular resistance by sympathetic nerve blockade is the main reason of hypotension. Relative dominance of parasympathetic system, activation of Bezold Jarish reflex (BJR) and increased baroreceptor activity may lead to bradycardia and some degree of hypotension. The responsible receptors for the BJR are mechanoreceptors located in the heart walls which participate in systemic responses to hyper and hypovolemia. They also include chemoreceptors sensitive to serotonin (5-HT3 receptors) (3-6).

Some animal and human studies illustrated that BJR can be decreased by 5-HT3 antagonists (7-10). On the other hand; serotonin (5-HT) is a critical thermoregulatory neurotransmitter. In non-anesthetized individuals, ondansetron, decreases core temperature attenuation that triggers shivering (11). The effect of two different doses of intravenous ondansetron on spinal-induced hemodynamic changes was considered as the primary outcome of this study and reduction in shivering during spinal anesthesia was considered as the secondary outcome.

## 2. Objectives

This study aimed to investigate the administration of intravenous ondansetron, a 5-HT3 receptor antagonist, which could attenuate spinal-induced hypotension, bradycardia and shivering.

## 3. Patients and Methods

This randomized double blinded clinical trial was performed in Dr. Shariati Hospital of Tehran University of Medical Sciences from November 2012 to March 2013. The study protocol was conformed to the ethical guidelines of the 1989 Declaration of Helsinki.

### 3.1. Ethics

Ethical approval for this study was provided by the Ethical Committee of Tehran University of Medical Sciences, Tehran, Islamic Republic of IRAN, protocol number 510, on October 20, 2012.

### 3.2. Allocation

Two hundred and ten patients aged 20-50 of American Society of Anesthesiologists (ASA) physical status, I or II -- scheduled for elective urologic, orthopedic or gynecologic surgeries under spinal anesthesia were enrolled in the study. The patients were instructed before the procedure and written informed consent was obtained individually before surgery. Randomization was done by computer-generated codes and was concealed until interactions were assigned. Patients with contraindications to spinal anesthesia, allergy to ondansetron or local anesthetics, history of hypertension, coronary artery disease or other cardiovascular diseases, and finally patients under any medication but study protocol drugs were excluded. Then the patients were divided into three equal groups: ondansetron 6 mg (n = 70), ondansetron 12 mg (n = 70) and control saline group (n = 70).

### 3.3. Intervention

All patients were premedicated by 10 mg oral chlordiazepoxide two hours before the operation. On arrival to the operating room, standard monitoring was applied to all patients, including pulse oximeter, electrocardiogram and noninvasive arterial blood pressure. Oxygen was delivered via a Venturi facemask at a rate of 4 L/min. An 18-gauge intravenous catheter was placed on the dorsum of non-dominant hand and patients received 5 ml/kg lactated Ringer solution warmed to 37˚C over 15 minutes before spinal anesthesia. The temperature of the operating room during the perioperative period was kept at a set average temperature of 24 ± 0.6˚C for all cases.

The patients in the control group (group C) received 20 mL normal saline. The intervention groups received 6 mg ondansetron (group A) or 12 mg ondansetron (group B), diluted in normal saline to the same volume. In all groups, solutions were infused over 5 minutes just before performing spinal anesthesia. All solutions were prepared by a resident of anesthesiology who was not involved in patient’s management or data collection. All patients were blocked in the lateral position and a 25 gauge needle inserted by midline approach into the L3-4 or L4-5 interspaces. After ensuring the correct position of the needle, 15 mg of 0.5% hypertonic bupivacaine was injected. Patients were immediately placed in the supine position after spinal block. The upper level of sensory blockade was evaluated by pinprick test from caudal to rostral direction at 5-min intervals up to 25 minutes. MAP and HR and the shivering were all recorded every 5 minutes up to 20 minutes. Shivering was graded according to the previous studies as the following scale:

Grade I; no shivering, Grade II; fasciculation in head and neck that was just visible as artifacts on ECG, Grade III; obvious tremor on head, neck and limbs, and Grade IV; generalized tremor throughout the body. Patients in grades I and II are assumed to have no shivering and grades III and IV denote shivering. In cases that MAP had dropped below 80 mm Hg or decreases more than 20%, 10 mg intravenous ephedrine would have been administered. When HR had dropped less than 50 beats per minute, 0.5 mg IV atropine would have been administrated. If patients had needed more sedation during surgery, 1 mg midazolam would have been given intravenously.

### 3.4. Statistical Analysis

Assuming a power of 95%, and a significance level of 5%, a sample size of 70 patients in each group would be sufficient to detect a 20% reduction in MAP among the groups. Statistical analysis was performed using SPSS package (version 17, SPSS, Chicago, IL). Normality of data distribution was tested by the Kolmogorov-Smirnov test. Hemodynamic responses were analyzed by repeated measures ANOVA (analysis of variance), demographic data were analyzed by 1-way ANOVA and chi-square test when appropriate. Two-tailed P value less than 0.05 was considered significant.

## 4. Results

**Figure 1.**
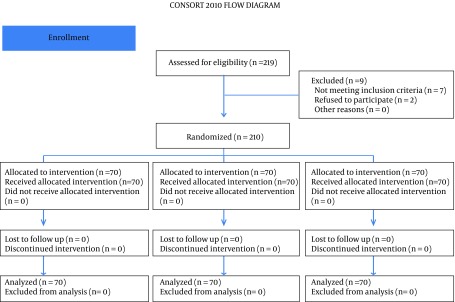
Subject Enrollment and Allocation in the Study Groups

**Figure 2.**
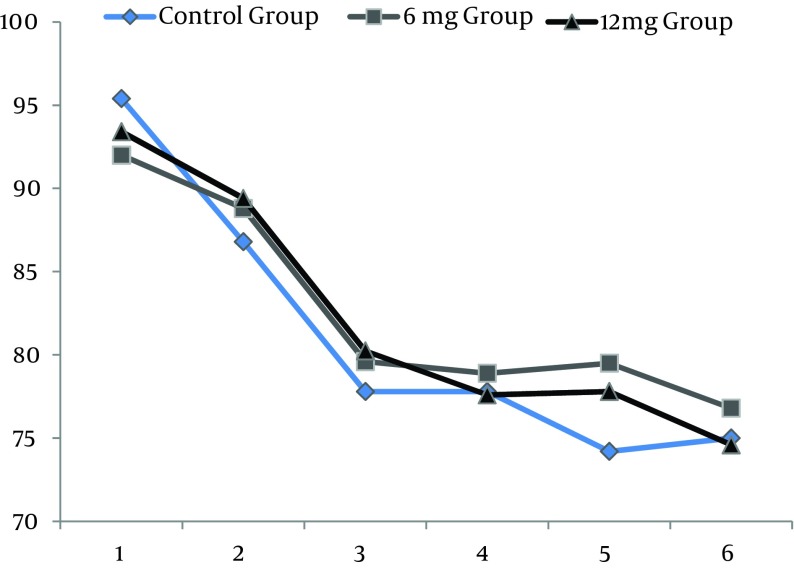
Comparing Mean Arterial Pressure Among the Study Groups During Different Measurements Times 1; 5 minutes before spinal anesthesia (SA), 2; immediately after SA, 3; 5 minutes after SA, 4; 10 minutes after SA, 5; 15 minutes after SA, 6; 20 minutes after SA.

**Figure 3.**
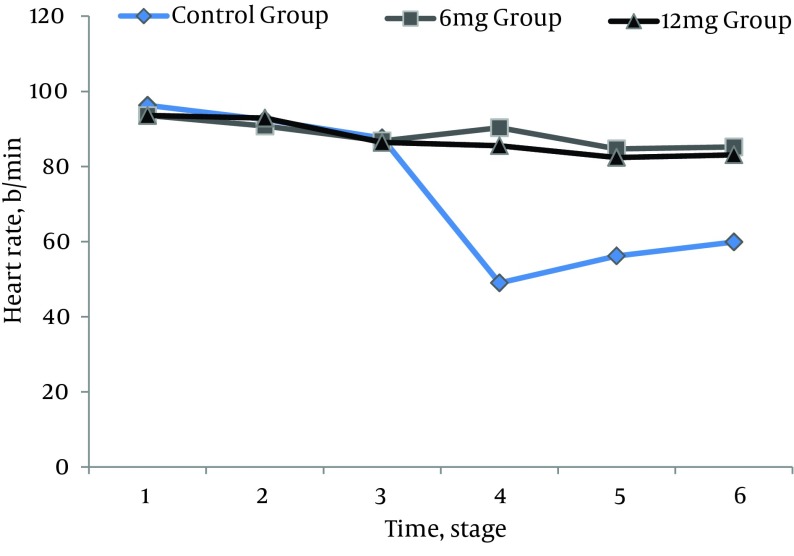
Comparing Heart Rate Among the Study Groups During Different Measurement Times 1; 5 minutes before spinal anesthesia, 2; immediately, after SA, 3; 5 minutes after SA, 4; 10 minutes after SA, 5; 15 minutes after SA, 6; 20 minutes after SA.

All 210 patients finished this study. All the spinal blocks were successful with a satisfactory level of anesthesia and all of the data were analyzed (Figure 1). There were no significant differences regarding demographic data (Table 1). Heart rates were statistically different between the ondansetron induced groups and control group. Ten patients (14%) in control group had HR < 50 bpm that required intravenous atropine (P =.02) (Figure 2). Mean arterial pressures were statistically different between the ondansetron induced groups and the control group. In the control group, 12 patients had MAP < 80 mm Hg and required vasopressors that was statistically significant compared to ondansetron induced groups (P = .04) (Figure 3). 

**Table 1.** Comparing Demographic Data Among the Study Groups O (n = 70) ^a^, ^b^, ^c^

Variable	Control	Ondansetron	
		6 mg	12 mg
**Age, y**	34.3 ± 10.1	34.4 ± 9.5	33.1 ± 7.9
**Sex**			
Male	44	45	43
Female	26	25	27
**Weight, kg**	84.1 ± 10.7	81.6 ± 12.3	79.9 ± 9.4
**ASA class**			
I	49	56	59
II	21	14	11

^a^ ASA: American Society of Anesthesiologists

^b^ Data are presented as Mean ± SD.

^c^ P > 0.05

**Figure 4.**
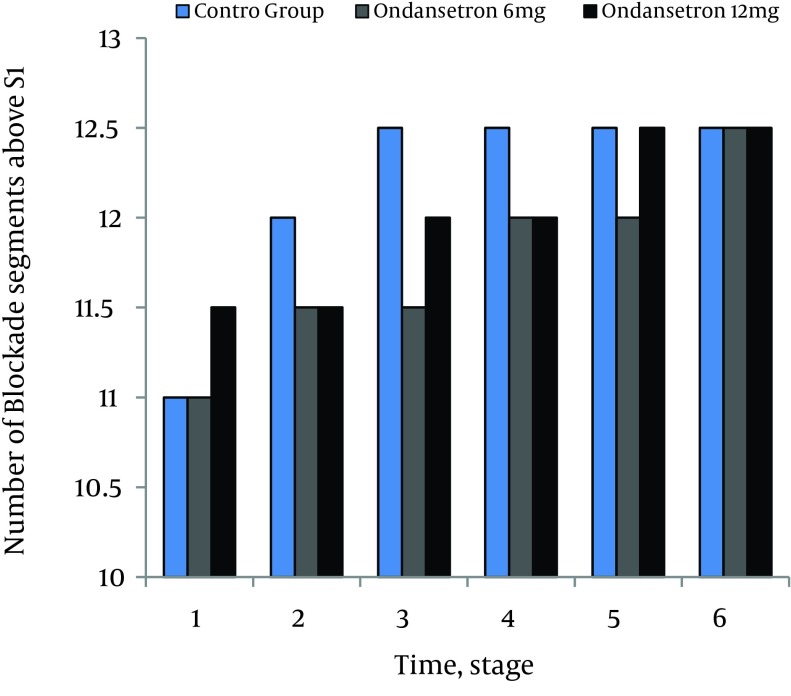
Comparison of Sensory Level Blockade Among the Study Groups During Different Measurements Times 1; immediately, after spinal anesthesia, 2; 5 minutes after SA, 3; 10minutes after SA, 4; 15 minutes after SA, 5; 20 minutes after SA, 6; 25 minutes after SA.

There were no significant differences in MAP and HR between the ondansetron groups (P = .06). None of the patients in both ondansetron groups experienced significant hypotension or bradycardia that required treatment. Incidences of shivering were 4% (3.70) in 6 mg and 2% (2.70) in 12 mg ondansetron induced groups respectively (P = .07). Incidence of shivering was 45% (32.70) in the control group that was statistically more than ondansetron groups (P = .02). Level of sensory blockades were not statistically different between the study groups (P = .07) (Figure 4). 

## 5. Discussion

This study demonstrated that administration of two different doses of intravenous ondansetron (6 mg and 12 mg) attenuates spinal induced hypotension, bradycardia and shivering compared to control group. There were no differences in hemodynamic profiles and shivering between the ondansetron groups. Hypotension during spinal anesthesia is a common side effect of this procedure, whereas bradycardia occurs rarely. Hemodynamic changes usually are benign; however, in selected patients, they may lead to serious consequences, including cardiac arrest, though it is a consequence of progressive bradycardia rather than progressive hypotension (4-6).

This complication results mainly from hyperactivity of the vagal nerve. It is worth emphasizing that the mechanisms of hypotension may be different from those producing severe bradycardia and cardiac arrest. 

While hypotension is due to a decrease in systemic vascular resistance and preload (which originates from sympathetic block, and blood redistribution), bradycardia is the consequence of the increase in baroreflex activity and BJR. Sympathovagal balance (parasympathetic system predomination) may provoke both kinds of side effects.

Methods to decrease the extent of cardiovascular consequences of spinal anesthesia include preloading with intravenous fluid, placing patients in positions facilitating venous return, and administration of vasopressor or atropine. On the other hand, Prophylactic administration of pharmacologic agents may be much more effective than hydration (1-6).

 Alpha-adrenergic agonists (phenylephrine) increase systemic vascular resistance (SVR) and blood pressure in this way but HR and cardiac output may reduce due to increase in afterload. Alpha and beta-adrenergic agonists, ephedrine, increase blood pressure, HR, cardiac output (CO) with slow increase in SVR. This difference in mechanism of action and physiological effect of both categories of drugs is used in the treatment of hypotension during spinal anesthesia (1-6).

In a study by Owczuk R et al. 71 patients operated under spinal anesthesia were allocated into the two equal groups. Intervention group received 8 mg of intravenous ondansetron compare to the saline control group prior to anesthesia. They concluded that ondansetron attenuated decrease in MAP and HR compared to control saline group that was similar to our study (10). In a study by Sahoo et al. (1) 52 parturients scheduled for elective caesarean section were randomly allocated into two groups. Before induction of spinal anesthesia Group O (n = 26) received intravenous ondansetron 4 mg; Group S (n = 26) received normal saline. Blood pressure, heart rate and vasopressor requirements were assessed. They concluded that intravenous ondansetron reduced hypotension and vasopressor use in this group of patients. Their finding was similar to ours except that in our study, we administrated two different doses of ondansetron. We had no significant differences in HR and MAP between the ondansetron groups.

In a study by Kalkaska et al. 75 patients undergoing spinal anesthesia were randomized into 3 groups. Group O and Group M were given 8 mg ondansetron and meperidine 0.4 mg/kg intravenously immediately before spinal anesthesia, respectively. Group C received saline at the same time. They concluded that ondansetron possess similar effects like meperidine in reducing shivering caused by spinal anesthesia. Their result was similar to our study, but we just compared two different doses of ondansetron with saline (not meperidine) (11). 

This finding could be crucially useful for risk population such as elderly patients who do not tolerate excess fluid infusion (due to cardiovascular decomposition) or pregnant women in whom the administration of vasoconstrictors can have adverse effects on uterine blood flow. Some earlier studies proposed that intravenous ondansetron may antagonize the sensory block of intrathecal local anesthetics. This could be a potential explanation for attenuation of hemodynamic changes following spinal anesthesia (12-16).

In our study, 10 minutes after spinal anesthesia MAP suddenly dropped from 90 to 50 mm Hg without any significant change in the HR. Hypotension without bradycardia following spinal anesthesia can be explained by the predominance of parasympathetic over sympathetic tone. These changes may also be related to antagonizing effect of ondansetron on the sensory block of intrathecal local anesthetics; however, we did not observe any significant changes in sensory block.

As the duration and type of the surgery, as well as blood loss and maintenance fluids could influence the results of such studies, further research is also recommended with a 5HT-3 receptor antagonists in different type of surgeries and comparing blood loses, and maintenance fluids. In conclusion, pretreatment with either 6 mg or 12 mg intravenous ondansetron reduces hemodynamic changes following spinal anesthesia without significant differences between these two doses of ondansetron.
